# Decision Tree Approach for Soil Liquefaction Assessment

**DOI:** 10.1155/2013/346285

**Published:** 2013-12-30

**Authors:** Amir H. Gandomi, Mark M. Fridline, David A. Roke

**Affiliations:** ^1^Department of Civil Engineering, The University of Akron, Akron, OH 44325, USA; ^2^Department of Statistics, The University of Akron, Akron, OH 44325, USA

## Abstract

In the current study, the performances of some decision tree (DT) techniques are evaluated for postearthquake soil liquefaction assessment. A database containing 620 records of seismic parameters and soil properties is used in this study. Three decision tree techniques are used here in two different ways, considering statistical and engineering points of view, to develop decision rules. The DT results are compared to the logistic regression (LR) model. The results of this study indicate that the DTs not only successfully predict liquefaction but they can also outperform the LR model. The best DT models are interpreted and evaluated based on an engineering point of view.

## 1. Introduction

Empirical classification techniques remain a highly researched topic, especially for real-world problems. In general, there are two types of classification techniques, statistical techniques and soft computing-based techniques. The most well-known soft computing techniques are artificial neural networks (ANNs), support vector machines (SVMs), and genetic programming (GP) [1]. These techniques are heuristic and mostly have a random nature; therefore, several runs are required to achieve useful results, even for a constant parameter setting. In contrast, statistical classifiers do not have a random nature and they are mathematically proven. The most common statistical techniques are logistic regression (LR) and decision tree (DT). Although DT techniques have been successfully applied to several real-world problems, they have been rarely used in engineering, especially geotechnical engineering (e.g., [2]).

Soil liquefaction is a process by which intergranular stresses vanish within the soil. Generally, loose and saturated sandy soils are susceptible to liquefaction. Seismic shaking, nonseismic vibration, or waved-induced shear stresses can cause dynamic liquefaction. The application of noncyclic shear stresses can cause static liquefaction in some loose sediments [3]. During the liquefaction process, a mass of saturated sandy soil tends to decrease in volume. If drainage conditions are not met or the drainage velocity is low, this decrease in volume will be accompanied with increased pore water pressure. Thereafter, the pore water pressure (*u*) will gradually equal the total stress (*σ*) in the soil. Then, the value of effective stress (*σ*′) becomes zero; consequently, shear strength (*τ*) approaches zero:

(1) $$\begin{array}{c} \sigma^{\prime }=\sigma -u,\\ \tau =\sigma^{\prime }\cdot \tan u. \end{array}$$

In such a case, the saturated sandy soil functions as a liquid and cannot bear any shear strength, resulting in flow of the sand [4].

The potential failure of critical structures due to liquefaction is a major concern in geotechnical engineering. Thus, extensive research has been conducted to understand the liquefaction phenomenon. The results obtained from in situ tests such as the standard penetration test (SPT) and the cone penetration test (CPT) are widely used for the evaluation of the soil liquefaction potential (e.g., [5, 6]). As the most common in situ test, SPT provides an approximate measure of the dynamic soil resistance, as well as a disturbed drive sample. In order to perform this test, a hollow thick-walled tube is driven into the ground and the number of blows to advance the split-barrel sampler a vertical distance of 300 mm is measured. Using a drop weight system, a 63.5 kg (140 lb) hammer is repeatedly dropped from 0.76 m to achieve three successive increments of 150 mm [7]. The *N* value (“blow count”) or SPT resistance is the sum of the number of blows to advance the second and third increments. SPT can be used for characterizing a wide range of soil types. CPT is another fast and economical in situ test that provides continuous profiling of geostratigraphy and soil property evaluation [7]. The test is performed by pushing a cylindrical steel probe into the ground at a constant rate of 20 mm/s and measuring the resistance to penetration. The standard penetrometer has a conical tip with 60° angle apex, 35.7 mm diameter body, and 150 cm^2^ friction sleeve. This test can be used in soils ranging from very soft clays to dense sands. CPT provides more accurate and reliable data for liquefaction analysis compared to more conventional soil tests, such as cyclic triaxial and simple shear tests. Thus, it can be considered as a very good complement to SPT measurements.

Classification techniques have been widely used for liquefaction modelling.  Table 1 shows the techniques and the soil test used in each of them. From the table, it is clear that soft computing techniques have been widely used for liquefaction modeling. However, statistical methods such as LR and DT have been only used for liquefaction assessment based on CPT data. This paper presents liquefaction assessments based on SPT data using both LR and DT.

Generally, statistical and soft computing techniques are not based on engineering fundamentals, as they are empirically developed based on experimental data. In the current study, we have forced the liquefaction assessment DTs to use SPT results as the first variable to corroborate with the engineering point of view. Another common problem of most classification techniques is that they are not interpretable. However, DT does not have this issue and its built models can be easily interpreted based on the nature of the parameters. In this study, three different DT methodologies are used, including chi-squared automatic interaction detection (CHAID), exhaustive CHAID (E-CHAID), and classification and regression tree (CART) algorithms. The DT results are further compared with logistic regression (LR) analysis as a classical benchmark.

## 2. Predictive Modeling Techniques

### 2.1. Decision Tree Algorithms

Decision trees are becoming a more attractive predictive modeling procedure because of the easy interpretation by nonstatisticians. One of the advantages of DT analysis is that the relationship between the binary dependent variable and the related independent variables is clearly illustrated using a tree structure. DT analysis is especially useful when the independent variables are expressed as both categories and continuous values. This nonparametric modeling procedure makes no assumptions about the underlying data. DT analysis determines how independent variables best combine to explain the outcome of a given binary dependent variable. In simple terms, DTs break down to “yes” or “no” statements according to “if-then” logic. In DT algorithms, the data set is partitioned into two or more mutually exclusive subsets in each split. The goal is to produce subsets of the data which are as homogeneous as possible with respect to the target (dependent) variable.

Kass [8] proposed the CHAID (chi-squared automatic interaction detection) algorithm as the first tree-based classification technique. Continuous predictors are discretized into several groups and changed to ordinal predictors. The main steps of the algorithm are the merging, splitting, and stopping steps. Merging is useful for any potential parent node that has more than two categories; in this step, potential parent nodes are merged until all adjusted *P* values are less than the defined *α*_merge_. Splitting of potential parents is the process used to find the best split, using the lowest *P* value; the splitting step selects which independent variable to be used to optimally split the node. The stopping step checks if the tree growing process should be stopped according to certain stopping rules (e.g., reaching the maximum tree depth level or minimum parent or child nodes).

Biggs et al. [9] proposed a new CHAID algorithm called exhaustive CHAID (E-CHAID). In E-CHAID, the basic CHAID algorithm is changed to improve merging and testing of dependent variables; as a consequence, E-CHAID is more computationally intensive. In the E-CHAID algorithm, there is no reference to any *α*_merge_ value; category merging continues until only two categories remain. Therefore, E-CHAID may not suitable for large data sets with many continuous predictor variables. The program then follows the splitting and stopping steps as described for the CHAID algorithm.

Breiman et al. [10] proposed the CART (classification and regression trees) algorithm. The main difference between the CART and CHAID algorithms is that the CART procedure will grow as a purely binary tree. Therefore, CART results are easier to understand, as parent nodes are always split into 2 child nodes. A complete binary tree algorithm partitions data and produces homogeneous subsets. In the CART procedure, we first develop the maximum tree and then prune it to avoid overfitting.

### 2.2. Logistic Regression

Logistic regression models are used when the dependent variable (*y*) is binary (e.g., the dependent variable can take the value of 1 with probability of success *π* or the value of 0 with probability of failure 1 − *π*), and the independent variables (*x*_*i*_) are either categorical or continuous values. The following is a representation of the binary multiple LR model:

(2) $$\begin{array}{c} \ln\left[\frac{\pi \left(x_{i}\right)}{1-\pi \left(x_{i}\right)}\right]=\ln\left[\frac{P\left(y_{i}=1/x_{i}\right)}{P\left(y_{i}=0/x_{i}\right)}\right]=\beta_{0}+\overset{p}{\underset{i=1}{\sum }}\beta_{i}x_{i}, \end{array}$$

where *β*_0_ is a constant and *β*_*i*_ are the coefficients of the independent variables in the model. Equation (2), called the likelihood function, is used for estimating the LR coefficients in the model. The maximum likelihood estimation method uses an iterative procedure to find the model coefficients that best match the pattern of observations in the sample data. Interpretation of the model comes from transforming the LR coefficients for each independent variable. Simply take the exponential of the coefficients (*e* ^*βi*^) to determine the influences of each independent variable on the dependent variable in terms of the odds ratio. To determine if each model coefficient is statistically significant, the Wald test will be used.

## 3. Liquefaction Modeling

### 3.1. Experimental Database and Data Preprocessing

A database [17] with 620 postearthquake observations and 12 variables has been used in this study. In addition to the seismic parameters, the database includes soil properties and the standard penetration test (SPT) results of the soil. The binary dependent variable is liquefaction and we selected a value of 0 for nonliquefied soils and a value of 1 for liquefied soils. The database includes the values of the following independent variables:

*Z*: soil specimen depth (m);
(*N*_1_)_60_: corrected PST number (%);
*F*_75_: percent fines less than 75 *μ*m (%);
*d*_*w*_: ground water table depth (m);
*σ*_vo_: total vertical stress (kPa);
*σ*_vo_′: effective vertical stress (kPa);
*a*_*t*_: threshold acceleration, which contributes to the strain-based procedure and along with strain-based safety factor indicates the exceedance of the threshold strain (Hanna et al. 2007) (g);
*τ*_av_/*σ*_vo_′: cyclic stress ratio, which illustrates the seismic demand on soil;
*V*_*s*_: shear wave velocity (m/s);
*φ*′: initial soil friction angle (°);
*M*_*v*_: earthquake magnitude (Richter);
PHA: peak horizontal acceleration at ground surface (g).

The database was randomly divided into training and testing subsets. The training data were used for the model development using the algorithms. The testing data were used to evaluate the generalization capability of the obtained models on the unseen data. In order to obtain a consistent data division, several combinations of the training and testing sets were considered. Out of the 620 records, 80% was used as the training data and the remaining 20% of the data sets were taken for the testing of the generalization capability of the DTs and LR.

The descriptive statistics of the data used in this study are given in Table 2. From this table, it can be seen that the database contains only high magnitude earthquakes. The records indicate that 256 sites were liquefied and the remaining 364 were not liquefied.

The correlation matrix is computed for all parameters and is presented in Table 3. As expected, the total and effective vertical stresses have strong correlation, and specimen depth correlates strongly with both stresses. From this table, SPT results have high correlation with initial soil friction angle. This is expected as the friction angle represents the shear capacity of soil, which is the most important parameter in SPT results.

### 3.2. Model Construction Using DTs

**Figure 1:**
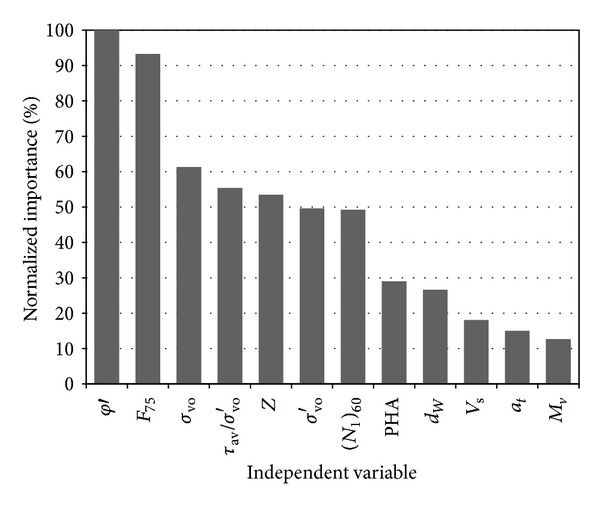
Importance of independent variables.

The procedure of the CART algorithm ranks the independent variables in terms of their predictive power; the CART algorithm therefore serves as a powerful exploratory tool for understanding the underlying structure of the data [27]. The relative importance of the 12 independent variables is shown in Figure 1. Figure 1 indicates that the most important predictor variable is friction angle (*φ*′). Therefore, the first split of the CART algorithm is based on friction angle. In the CHAID and E-CHAID algorithms the lowest adjusted *P* value in each level determines the most associate independent variable. After calculation, the friction angle has the lowest adjusted *P* value at the beginning of the CHAID and E-CHAID algorithms, and again serves as the first split.

Based on geotechnical engineering theory, the SPT test results ((*N*_1_)_60_) should be the most associated parameter and should therefore be the first split in the DT algorithms. Therefore, for DT modeling, each algorithm was implemented in two ways:

1. let the DT algorithm determine the first split based on its own statistical method;
2. force the DT algorithm to use SPT test results as its first split.

The performance of a DT model mainly depends on the architecture and parameter settings. There are many parameters that are involved in the DT algorithms. For the CHAID and E-CHAID algorithms, the choices of maximum tree depth and minimum number of cases in the parent and child nodes play an important role in model construction. After evaluating different values for the maximum tree depth, it is apparent that only training results (not the results for unseen data) improve for maximum depths greater than 4. Therefore, to avoid overfitting, we have set the maximum tree depth as 4. Products of ten are considered for the minimum number of cases in the parent nodes; the minimum number of cases in the child nodes is always set as half of the parent nodes' minimum. For this study, 30 and 15 are set as the values for the parent and child node maximum number of cases. Two chi-square statistics are commonly used in the CHAID and E-CHAID algorithms: Pearson and likelihood ratio. Pearson is faster than the likelihood ratio and is more suitable for very large databases. The likelihood ratio is more robust than Pearson, but its calculation is more time consuming and it is therefore more suitable for a small database [28]. Due to its robustness, the likelihood ratio is used in this study as the chi-square statistic for both the CHAID and E-CHAID algorithms.

For the CART algorithm, the minimum number of cases in the parent and child nodes is set just like the other DT algorithms. The maximum tree depth is set as 7 and pruning is used to avoid overfitting (pruning at least one level in this problem). IBM SPSS software package version 21 [29] was used to perform the analysis.

### 3.3. Model Construction Using LR

In the conventional classification process, logistic regression analysis is an important tool for building a model. In this study, the LR algorithm was performed to approximate the predictive power of the DT techniques in comparison with another statistical classification technique.

The LR models cannot be developed using the same input variables and data divisions as DTs. Before finding the final LR model, any multicollinearity problems between the independent variables were solved by removing the highly correlated independent variables from the model. Severe multicollinearity can cause instability in the model coefficients, especially when highly correlated variables are included in the model.

## 4. Results and Discussion

As described above and indicated in Table 4, six different DT-based models were obtained for the assessment of the soil liquefaction. The DT models for which the SPT results were the first variable are indicated with the suffix “SPT.” Overall performance of the DT and LR-based models in terms of misclassification rate (risk) on the training data and testing data subsets and the full data set are summarized in Table 4.

Two important characteristics of a classification model are sensitivity and specificity. Sensitivity quantifies the proportion of correctly identified positives (liquefied soils) and specificity gives the proportion of correctly identified negatives (nonliquefied soils). Sensitivity and specificity of all DT and LR models are also presented in Table 5. Comparing the performance of DTs and LRs, as tabulated in Tables 4 and 5, the DT results are clearly better than the LR results: the DTs generally have lower risk values and higher sensitivity and specificity values on the training, testing, and complete datasets. An interesting point from these tables is that when the DT algorithms are forced to used SPT results as the first variable, the model performances on the testing datasets (unseen data) are always improved. This suggests that the strategy of using SPT as the first variable is very effective for future prediction of new datasets. However, as expected, if the DTs use their own criteria for choosing the first variable during the training process, performance is improved on the training datasets but not the testing datasets.

As shown in Table 4, CHAID models (CHAID and CHAID-SPT) produce the lowest risks among all DTs and LRs. CHAID-SPT has the best performance on unseen testing data sets (risk equal to 0.169) and regular CHAID has the best performance on the training dataset (risk equal to 0.141). As predicting liquefied soil after earthquakes is very important, the highest sensitivity value for each set is bolded in Table 5. From this table, it can be seen that CHAID-SPT has the highest sensitivity on the testing datasets (sensitivity equal to 0.867) and CHAID has the highest sensitivity on the training datasets (sensitivity equal to 0.806). Therefore, these two models have been chosen as the final models.

**Figure 2:**
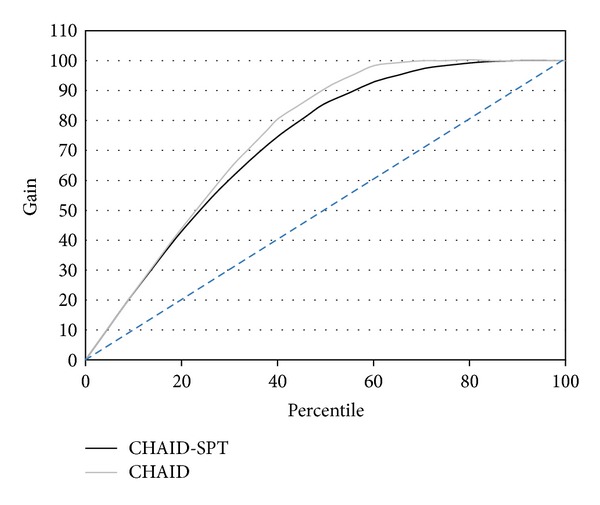
Gain plot of the CHAID and CHAID-SPT models.

Percentage of total records in the target category in each node is recognized as gain. The gains plot is a line chart of cumulative percentile gains calculated using cumulative percentile target over total target [28]. The gains plot of the selected models (CHAID and CHAID-SPT) for the training results is presented in Figure 2. In this figure the straight line shows the random selection model, and the area between it and the model curve shows the model benefit area. Figure 2 shows the effectiveness of the CHAID and CHAID-SPT models. From this figure, it is clear that the CHAID model has the better benefit on the training data.

**Figure 3:**
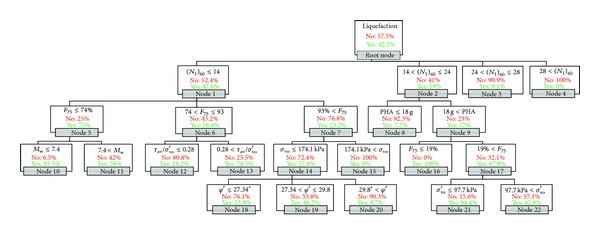
Final CHAID-SPT tree.

The final decision tree for the CHAID-SPT model is presented in Figure 3. This tree has 22 nodes, 14 of which are terminal nodes. The first level of the tree was split into four initial branches, corresponding to four ranges of SPT values. As seen in the first level of the tree, the two nodes for the highest SPT values are terminal nodes, which are interesting and show the importance of the SPT variable. For these two nodes (nodes 3 and 4), the chance of liquefaction is very low, which corresponds with geotechnical engineering theory.

In the second level, the percentage of fines is the best predictor for the soils with the lowest SPT values and its three splits show that liquefaction probability decreases with increasing percentage of fines. For larger SPT values (14 < SPT ≤ 24), peak ground surface horizontal acceleration is the best predictor. In this range, increasing peak horizontal acceleration substantially raises the chance of liquefaction, which corresponds with earthquake engineering fundamentals.

The third level consists largely of terminal nodes and has branches based on four different variables: earthquake magnitude, cyclic stress ratio, total vertical stress, and percentage of fines. In this level, the liquefaction probability decreases with increasing total vertical stress and increasing cyclic stress ratio. Like in the second level, liquefaction probability decreases with increasing percentage of fines.

In the fourth level, there are five terminal nodes: one node is split into three ranges of soil friction angle and one node is split using two ranges of effective vertical stress. In this level, the liquefaction probability decreases with increasing effective vertical stress, which corresponds to the trend of total vertical stress in the third level and geotechnical engineering fundamentals.

**Figure 4:**
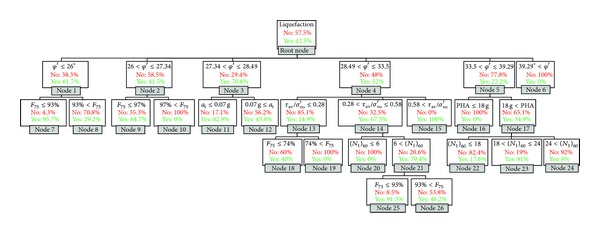
Final CHAID tree.

The final decision tree for the CHAID model is shown in Figure 4. This tree has more terminal nodes than the CHAID-SPT tree, with 26 nodes (17 of which are terminal nodes). The first level of the tree was split to six initial branches according to the initial soil friction angle, which was indicated as the variable most associated with liquefaction, according to the CHAID algorithm. As seen in the first level of the tree, the node for the highest angles (*φ*′ > 39.29°) is a terminal node that indicates zero probability of liquefaction.

In the second level, nodes 1 and 2, related to the lowest initial soil friction angle (*φ*′ ≤ 27.34°), are split using the percentage of fines. This indicates that percentage of fines is most associated with liquefaction prediction for low soil friction angles. From the branches of the nodes 1 and 2 of the CHAID tree (in the third level), the liquefaction probability decreases with increasing percentage of fines, which agrees with the previous results. The soil with an initial friction angle between the 28.49° and 33.5°, the cyclic stress ratio is the best predictor of liquefaction. The probability of liquefaction increases significantly with an increasing cyclic stress ratio.

In the third level, only one node is a parent node; the other six nodes are terminal nodes. In the fourth level, there are only two terminal nodes split using the percentage of fines. From the third and forth levels, it can be noted that liquefaction probability decreases with increasing percentage of fines.

From a comparison of the CHAID-SPT and CHAID trees, it can be seen that percentage of fines is the best second-level predictor for the lowest SPT values ((*N*_1_)_60_ ≤ 14) and for the lowest initial soil friction angle values (*φ*′ ≤ 27.34°). Another similarity of the developed CHAID and CHAID-SPT trees in the second level is that peak horizontal acceleration is the best predictor for midrange values of SPT (14 < (*N*_1_)_60_ ≤ 24) and midrange values of soil friction angle (33.5° < *φ*′ ≤ 39.29°). In the second level, for the highest values of SPT and initial soil friction angle, there is zero liquefaction probability. From these similarities, there appears to be a strong correlation between SPT and initial soil friction angle. This general conclusion can be also verified with the correlation coefficient of these two parameters (presented in Table 3). From these data, SPT and initial soil friction angle are high correlated parameters.

## 5. Summary and Conclusions 

Six decision tree models were successfully developed in this study to classify the liquefied and nonliquefied soil using three different techniques. Based on geotechnical engineering theory, three of the decision trees were developed using SPT as the first variable. The results of the DT models were compared with those of an LR model; the results show that DT models generally have better performance than the LR model for this problem.

The two best models (CHAID and CHAID-SPT) were chosen based on the risk values and the sensitivity of the liquefaction prediction. The CHAID-SPT tree has better performance on the unseen (testing) dataset, indicating that basing DT models on engineering fundamentals can improve model performance. The general interpretation of the trees corresponds well with engineering theory, which indicates the reliability of the models. From the interpretation results of the trees, the SPT values and initial soil friction angle values (used as first variables of the trees) have strong correlation.

Some terminal nodes have small sample sizes, which may lead to results that are anecdotal. However, these anecdotal conclusions about soil liquefaction assessment are highly informative from an exploratory point of view. As the earthquake parameters do not have a wide range, their interpretation may not be accurate enough. Therefore, for future research, the database used in this study should be expanded to a wider range of earthquake parameters.

## Figures and Tables

**Table 1:** Different liquefaction models proposed in the literature.

Type of technique	Reference	Year	Soil test	Classification technique	Number of records
Soft computing	[11]	1994	SPT	ANN	85
	[12]	1996	CPT	ANN	109
	[13]	1998	SPT	ANN	105
	[14]	2002	CPT	ANN	170
	[15]	2006	CPT and SPT	SVM	109 and 85
	[16]	2007	CPT and SPT	SVM	170 and 105
	[17]	2007	SPT	ANN	620
	[18]	2007	CPT	SVM	226
	[19]	2009	CPT	ANN	226
	[20]	2011	CPT	GP	170
	[21]	2011	SPT	GP and ANN	569
	[22]	2012	CPT	GP	170
	[23]	2013	CPT	GP	170
	[24]	2013	CPT	GP	170

Statistical	[25]	2006	CPT	LR	396
	[26]	2008	CPT	DT	178

**Table 2:** Descriptive statistics of variables used in the model development.

Predictor	Minimum	Mean	Maximum	Standard deviation
*Z*	0.8	7.66	19.8	4.90
(*N*_1_)_60_	1	14.48	75	11.39
*F* ≤ 75 *μ*m	1	62.99	100	34.28
*d*_*w*_	0.35	1.45	10	1.20
*σ*_vo_	12.1	144.60	408.9	98.20
*σ*′_vo_	7.5	82.48	233.7	52.84
*a*_*t*_	0	0.07	0.85	0.07
*τ*_av_/*σ*_vo_′	0.12	0.37	0.77	0.15
*V*_*s*_	37	166.98	500	67.09
*φ*′	23.46	31.96	52.08	4.85
*M*_*v*_	7.4	7.49	7.6	0.10
PHA	0.18	0.38	0.67	0.15

**Table 3:** Correlation matrix.

	*Z*	(*N*_1_)_60_	*F*_75_	*d*_*w*_	*σ*_vo_	*σ*_vo_′	*a*_*t*_	*τ*_av_/*σ*_vo_′	*V*_*s*_	*φ*′	*M*_*v*_	PHA	Liq.
*Z*	1	0.39	−0.25	0.08	1.00	0.98	−0.23	−0.12	0.58	0.53	0.49	−0.11	−0.30
(*N*_1_)_60_	0.39	1	−0.57	0.08	0.41	0.42	0.03	0.15	0.40	0.85	0.15	0.17	−0.27
*F*_75_	−0.25	−0.57	1	−0.18	−0.27	−0.30	−0.12	−0.07	−0.30	−0.54	−0.28	−0.16	−0.06
*d*_*w*_	0.08	0.08	−0.18	1	0.07	0.21	0.14	−0.27	0.16	0.13	0.21	−0.02	−0.13
*σ*_vo_	1.00	0.41	−0.27	0.07	1	0.99	−0.22	−0.09	0.59	0.55	0.50	−0.07	−0.29
*σ*_vo_′	0.98	0.42	−0.30	0.21	0.99	1	−0.19	−0.10	0.61	0.56	0.55	−0.03	−0.28
*a*_*t*_	−0.23	0.03	−0.12	0.14	−0.22	−0.19	1	−0.06	0.44	−0.02	0.00	0.11	−0.08
*τ*_av_/*σ*_vo_′	−0.12	0.15	−0.07	−0.27	−0.09	−0.10	−0.06	1	−0.03	0.09	−0.20	0.90	0.25
*V*_*s*_	0.58	0.40	−0.30	0.16	0.59	0.61	0.44	−0.03	1	0.47	0.32	0.04	−0.21
*φ*′	0.53	0.85	−0.54	0.13	0.55	0.56	−0.02	0.09	0.47	1	0.29	0.12	−0.35
*M*_*v*_	0.49	0.15	−0.28	0.21	0.50	0.55	0.00	−0.20	0.32	0.29	1	−0.11	−0.15
PHA	−0.11	0.17	−0.16	−0.02	−0.07	−0.03	0.11	0.90	0.04	0.12	−0.11	1	0.19
Liq.	−0.30	−0.27	−0.06	−0.13	−0.29	−0.28	−0.08	0.25	−0.21	−0.35	−0.15	0.19	1

**Table 4:** Risk estimation of the DT and LR models.

Model	Testing	Training	All
CHAID	0.234	**0.141**	**0.160**
E-CHAID	0.234	0.161	0.176
CART	0.194	0.163	0.169
CHAID-SPT	**0.169** ^a^	0.188	0.184
E-CHAID-SPT	0.177	0.161	0.165
CART-SPT	0.185	0.165	0.169
LR	0.258	0.270	0.268

^a^Bold sets are the lowest risk values for each set.

**Table 5:** Sensitivity and specificity of the DT and LR models.

Model	Testing		Training		All	
	Sensitivity	Specificity	Sensitivity	Specificity	Sensitivity	Specificity
CHAID	0.622	0.848	**0.806**	0.898	0.773	0.887
E-CHAID	0.644	0.835	0.754	0.902	0.734	0.887
CART	0.667	0.886	0.739	0.909	0.727	0.904
CHAID-SPT	**0.867** ^a^	0.810	0.763	0.849	0.781	0.841
E-CHAID-SPT	0.711	0.886	0.768	0.891	0.758	0.890
CART-SPT	0.800	0.823	0.796	0.863	**0.797**	0.854
LR	0.822	0.696	0.673	0.772	0.699	0.755

^a^Bold sets are the highest sensitivity values for each set.
